# Evaluating mating compatibility within fruit fly cryptic species complexes and the potential role of sex pheromones in pre-mating isolation

**DOI:** 10.3897/zookeys.540.6133

**Published:** 2015-11-26

**Authors:** M. Laura Juárez, Francisco Devescovi, Radka Břízová, Guillermo Bachmann, Diego F. Segura, Blanka Kalinová, Patricia Fernández, M. Josefina Ruiz, Jianquan Yang, Peter E.A. Teal, Carlos Cáceres, Marc J.B. Vreysen, Jorge Hendrichs, M. Teresa Vera

**Affiliations:** 1 Cátedra Terapéutica Vegetal, Facultad de Agronomía y Zootecnia (FAZ), Universidad Nacional de Tucumán (UNT), San Miguel de Tucumán; Argentina; 2 Consejo Nacional de Investigaciones Científicas y Técnicas (CONICET), Argentina; 3 Instituto Nacional de Tecnología Agropecuaria (INTA), Hurlingham, Argentina; 4 Institute of Organic Chemistry and Biochemistry, Prague, Czech Republic; 5 Fujian Agriculture and Forestry University, China; 6 Chemistry Research Unit, USDA-ARS, Gainesville, USA; 7 Insect Pest Control Laboratory (IPCL), Joint FAO/IAEA Division of Nuclear Techniques in Food and Agriculture, Vienna, Austria; 8 Insect Pest Control Section, Joint FAO/IAEA Division of Nuclear Techniques in Food and Agriculture, Vienna, Austria

**Keywords:** species delimitation, field cages, Tephritidae, *Anastrepha fraterculus*, *Bactrocera dorsalis*, ﻿ *Ceratitis fasciventris*, *Ceratitis anonae*, ﻿ *Ceratitis rosa*

## Abstract

The study of sexual behavior and the identification of the signals involved in mate recognition between con-specifics are key components that can shed some light, as part of an integrative taxonomic approach, in delimitating species within species complexes. In the Tephritidae family several species complexes have received particular attention as they include important agricultural pests such as the *Ceratitis fasciventris* (Bezzi), *Ceratitis anonae* (Graham) and *Ceratitis rosa* Karsch (FAR) complex, the *Bactrocera dorsalis* (Hendel) complex and the *Anastrepha fraterculus* (Wiedemann) complex. Here the value and usefulness of a methodology that uses walk-in field cages with host trees to assess, under semi-natural conditions, mating compatibility within these complexes is reviewed, and the same methodology to study the role of chemical communication in pre-mating isolation among *Anastrepha fraterculus* populations is used. Results showed that under the same experimental conditions it was possible to distinguish an entire range of different outcomes: from full mating compatibility among some populations to complete assortative mating among others. The effectiveness of the methodology in contributing to defining species limits was shown in two species complexes: *Anastrepha fraterculus* and *Bactrocera dorsalis*, and in the case of the latter the synonymization of several established species was published. We conclude that walk-in field cages constitute a powerful tool to measure mating compatibility, which is also useful to determine the role of chemical signals in species recognition. Overall, this experimental approach provides a good source of information about reproductive boundaries to delimit species. However, it needs to be applied as part of an integrative taxonomic approach that simultaneously assesses cytogenetic, molecular, physiological and morphological traits in order to reach more robust species delimitations.

## Relevance of studying sexual behavior and mating compatibility in complexes of pest species

The biological species concept proposes the occurrence of reproductive isolation barriers that prevent interbreeding and hybridization between species (Mayr 1942, Dobzhansky 1970). According to Dobzhansky (1937, 1970), reproductive isolation can take place in different forms and can be classified as pre-zygotic barriers that act before fertilization, and as post-zygotic barriers that act after fertilization or even after the formation of hybrids. Pre-zygotic barriers comprise those that prevent mating (pre-mating) such as behavioral (also referred as sexual, but see Coyne and Orr (2004) for a discussion), ecological (i.e. habitat, temporal and pollinator isolation) and mechanical isolation barriers, and those that prevent gene flow and occur after mating (post-mating), such as copulatory behavioral isolation, cryptic female choice (Eberhard 1996, 2015) and gametic isolation. Post-zygotic barriers can be extrinsic and related to the environment (i.e. ecological inviability and behavioral sterility) or intrinsic reflecting developmental problems in the hybrids independently of the environment (i.e. hybrid inviability and sterility) (Coyne and Orr 2004). Determining the evolutionary forces that lead to speciation and the mechanisms (i.e. type of reproductive barriers) by which populations initially diverge and are kept isolated afterwards is one of major challenges in evolutionary biology and speciation studies (Marie Curie SPECIATION Network 2012). Behavioral isolation, which includes every behavior or signaling trait that affects recognition between con-specific mates, attractiveness and mate choice (Panhuis et al. 2001), has been considered a key component in speciation initiation (Coyne and Orr 2004).

The elucidation of the mechanisms that underlie reproductive incompatibility among species within cryptic species complexes is relevant to accurately delimit species and to understand mate recognition systems. According to Bickford et al. (2007), cryptic species are defined as “two or more distinct species that are erroneously classified (and hidden) under one species name”. Following the biological species concept, demonstrating the occurrence of reproductive incompatibility should be sufficient to delimit species (Mayr 1942); however this is not universally accepted (see de Queiroz 2005). The current recommendation is to use integrative taxonomy and combine evidence from molecular, behavioral, physiological and morphological traits (Dayrat 2005). Schlick-Steiner et al. (2010) suggest that at least three different, independent criteria should be used to delimit species. Delimiting species is particularly relevant in those cases where clear taxonomic identification and species delimitation have implications for biogeography, conservation, international trade, and pest management strategies.

One paradigmatic example of the need to resolve species complexes comes from Tephritidae fruit flies. This family is composed of approximately 5,000 species of fruit flies (Norrbom et al. 1999, 2004), some of which are destructive pests of fruit and vegetable production (White and Elson-Harris 1994). Some major pest species complexes include taxonomically described “species” that are in fact geographical variants of the same species. Other complexes are composed of populations that are taxonomically grouped within the same pest species but display different biological and genetic traits and show reproductive isolation which is strong evidence that they are indeed different species. The uncertain taxonomic status has at least two important practical implications. The first is related to fruit commercialization as international movement of fruits and vegetables is conditioned by pest presence in the exporting and importing countries. The second is related to pest management. Several species within this family are managed using area-wide integrated pest management (AW-IPM) (Klassen and Curtis 2005, Vreysen et al. 2007) approaches that include a sterile insect technique (SIT) component (Knipling 1955). The SIT is based on the repeated release in the targeted area of mass-reared male flies of the pest species that are sterilized by using ionizing radiation. Wild females that mate with sterile males are inseminated with sterile sperm that induces embryonic lethality; therefore they do not produce offspring or produce offspring that is unviable. The resulting reduction in population replacement will cause a gradual decline in the population size of the targeted species (Dyck et al. 2005). Sexual compatibility between females from the target wild population and the released sterile males is absolutely essential for the SIT to be an effective control method.

Here we aimed to review the value and usefulness of a methodology that assess mating compatibility under standard semi-natural conditions. We focused on the Tephritidae family in general, and on the *Anastrepha fraterculus* (Wiedemann) cryptic species complex and the *Bactrocera dorsalis* complex (Drew and Hancock 1994) in particular. In addition, the use of walk-in field cages was evaluated as a method for studying the role of chemical communication in pre-mating isolation within the *Anastrepha fraterculus* cryptic species complex.

## Mating compatibility field cage tests in the Tephritidae

The international manual for Product Quality Control for Sterile Mass-Reared and Released Tephritid Fruit Flies (FAO/IAEA/USDA 2014) provides a standard Mating Performance Field Cage Test to be carried out under semi-natural conditions in walk-in field cages (Box 1). The protocol has improved over the years, within the framework of SIT applications, and provides information on the mating behavior of the used strains and insect populations, with particular focus on mating competitiveness and mating compatibility. In the case of mating competitiveness tests, the goal is to evaluate the ability of sterile males to compete with wild males for mating with the wild females of the target population. In the case of mating compatibility, the goal is to determine the degree of sexual compatibility between the target wild population and any available strain already adapted to laboratory mass-rearing conditions. The compatibility should be measured before any large-scale rearing or release operations are initiated, while the competitiveness needs to be assessed at regular intervals during implementation of such a program. Cases of unsatisfactory male competitiveness or even mating incompatibility should either lead to the selection of a different strain or the colonization of a new strain from the target area.

**Box 1.** Mating compatibility using walk-in field cage test procedures

The standardized Mating Performance Field Cage Test (FAO/IAEA/USDA 2014) involves the release of males and females from two different populations into a cylindrical cage (2 m tall and 3 m in diameter) (Calkins and Webb 1983). The cage contains one tree that provides a courting and mating substrate for the flies. Prior to the experiments, flies are sorted by sex on the day of emergence, the sexes kept in separate rooms, and provided with water and food until sexual maturation. Releasing flies from two different populations in a field cages requires a marking system to make them distinguishable. This can be achieved either with a dot of water-soluble paint applied on the notothorax, feeding the adults with a diet that contains a food colorant or with fluorescent dyes applied to the pupae before adult emergence. The colors should be applied randomly for each replicate to avoid any potential interference of the color in mate selection. On the day of the test, males from the two selected populations are released into the field cage. After the release, males are given time to acclimatize (15–30 min), establish territories and initiate their sexual displays. The time of the day to initiate the test depends on the temporal sexual activity pattern of the populations under investigation. Females are released after males begin to display behaviors related to sexual activity. An observer, who is located inside the field cage, screens the tree and the inside of the cage (netting, poles) and searches for mating couples. Once a couple is detected, the observer removes the couple by gently coaxing it into a small vial and records the origin or type of the male and the female, the time of onset of copulation, and the location of the couple, i.e. on the poles of the cage, the cage netting or in the tree. The location on the tree can be further specified in terms of height (upper, middle or low), cardinal axes (north, east, south or west), and location within the canopy (in the peripheral layer of the canopy or in the core of the canopy). The vial is kept inside the cage (placed under the shadow of the tree) until the couple disengages and the time at which copulation ends is recorded. The test is completed when sexual activity ceases. Only those experiments in which at least 20% of the males and females from each strain engaged in matings are considered as part of the data set and a minimum of nine replicates are required for each treatment of the field cage test. The data are used to derive several mating indices such as the Relative Isolation Index (RII) (McInnis et al 1996), the Stalker’s Index (I) (Stalker 1942), and the Index of Sexual Isolation (ISI) (Cayol et al. 1999) that provide indications on the level of mating compatibility. The ISI ranges from -1 to 1 and when the confidence interval includes zero, the tested combinations are considered as sexually compatible. In addition, the Male and Female Relative Performance indices (MRPI and FRPI, respectively) (Cayol et al. 1999) estimate the relative participation of each sex of a given population (regardless of the origin of its partner). The comparison of the time to start copula for each particular mating combination (latency), the time spent *in copula* and the location of the couples provide additional cues to better understand possible isolation mechanisms. The combined analysis of the different indices (ISI, MRPI and FRPI) and the other recorded variables (or even a more detailed analysis of courtship components and other behaviors, see Briceño and Eberhard 1998, Lux et al. 2002) provide a complete and reliable description of the sexual compatibility between populations (FAO/IAEA/USDA 2014).

Mating Performance Field Cage Tests can be applied to assess mating compatibility among two or more populations/species from a given species complex in studies aiming to clarify the taxonomic relationships within species complexes. Data derived from these tests can generate simple, reproducible, meaningful indices of sexual compatibility that can be used to make comparisons between different populations by observing the components of the courtship and mating behavior and any other intra- and inter-sexual interactions during the time of sexual activity. Mating compatibility studies using this standard test gave relevant results for different fruit fly species, including *Ceratitis capitata* (Wiedemann), species within the FAR complex, species within the *Bactrocera dorsalis* complex, *Zeugodacus cucurbitae* (Coquillett) and the *Anastrepha fraterculus* cryptic species complex.

For *Ceratitis capitata*, Cayol et al. (2002) studied the mating compatibility among eight populations that originated from different geographical areas of the world, i.e. Australia, France (La Réunion island), Greece, Guatemala, Israel, Kenya, Portugal (Madeira island) and South Africa. The authors concluded that there was no evidence of mating incompatibility and in addition, full compatibility was shown between these populations and four genetic sexing strains (Franz 2005) with a different genetic background. These results proved that only very few genetic sexing strains are needed across the world when applying the SIT against *Ceratitis capitata*. These data are consistent with Lux et al. (2002) who analyzed in detail the courtship behavior of several wild populations and mass-reared strains of *Ceratitis capitata* and found no qualitative or quantitative differences.

The *Ceratitis* FAR complex is composed of three species, *Ceratitis anonae* (Graham), *Ceratitis rosa* Karsch and *Ceratitis fasciventris* (Bezzi) that occur in certain areas of Africa. Due to their highly invasive potential and some difficulties in distinguishing some members of the complex morphologically, a number of different approaches for species recognition were used (reviewed in De Meyer et al. 2015). An in-depth molecular study (Virgilio et al. 2013) revealed greater genetic differentiation than expected at the intra-species level. In particular, the analysis showed the presence of five genotypic groups, involving two *Ceratitis rosa*, two *Ceratitis fasciventris* and one *Ceratitis anonae*, suggesting that *Ceratitis rosa* and *Ceratitis fasciventris* may each include more than one species. In contrast, *Ceratitis anonae* did not show a clear intra-specific population genetic structure. Two different *Ceratitis rosa* populations, lowland and highland, with different biological attributes such as developmental rates were characterized along an altitudinal transect (Mwatawala et al. 2015). Recently, a field cage mating compatibility study using *Ceratitis rosa* and *Ceratitis fasciventris* showed that the level of isolation between low and highland populations of *Ceratitis rosa* was as high as that found between the two species, irrespective of whether the *Ceratitis rosa* populations being compared with *Ceratitis fasciventris* originated from the low- or the highlands (S. Ekesi pers. comm.). These significant levels of mating isolation could be correlated not only with the strong population structuring found at the molecular level (Virgilio et al. 2013) but with the differences found in pheromones and cuticular hydrocarbons (CHCs) (Vaníčková et al. 2014). In all, the evidence gathered confirmed cryptic speciation and the existence of more than three species within the FAR complex (De Meyer et al. 2015).

A whole body of evidence has been collected that supports the hypothesis that the nominal species *Bactrocera dorsalis* (Hendel), *Bactrocera papayae* Drew and Hancock, and *Bactrocera philippinensis* Drew and Hancock, all belonging to the *Bactrocera dorsalis* complex, are in fact one biological species with geographical variation. Schutze et al. (2013) evaluated for the first time the mating compatibility among all possible combinations of these members using the Mating Performance Field Cage Test and found no mating isolation among *Bactrocera dorsalis*, *Bactrocera papayae* and *Bactrocera philippinensis*. In contrast when any of these entities were tested with *Bactrocera carambolae*, a certain degree of pre-mating isolation was detected. This was not only revealed by the differences in the numbers of mating types, but also in the location of the couples in the field cage: most couples involving *Bactrocera carambolae* females were found in the tree and those involving *Bactrocera papayae* and *Bactrocera dorsalis* females were found against the roof of the field cage. Schutze et al. (2013) concluded that *Bactrocera dorsalis*, *Bactrocera papayae* and *Bactrocera philippinensis* belonged to a single species. Similar mating compatibility studies between *Bactrocera invadens* from Kenya and *Bactrocera dorsalis* from China and Pakistan were carried out by Bo et al. (2014) using the same field cage methodology. Full mating compatibility was demonstrated also between both *Bactrocera dorsalis* populations and *Bactrocera invadens*, and hybrid offspring obtained between both crosses were viable for several generations. These results, in combination with findings from another field cage mating study (Chinvinijkul et al. 2015), as well as morphological, molecular genetics, cytological, sexual compatibility and chemo-ecology studies carried out over the past 20 years, led to the conclusion that these members of the *Bactrocera dorsalis* complex belonged to the same single species and their synonymization was recently published (Schutze et al. 2015a and references therein). Only *Bactrocera carambolae* was maintained as a valid biological species.

The different host use pattern of *Zeugodacus cucurbitae* observed in Mauritius and some African locations suggested the possibility of a sibling species complex, i.e. species that are the closest relative of each other and have not been distinguished from one another taxonomically (Bickford et al. 2007). If this were true, the use of the genetic sexing strain developed at the United States Department of Agriculture, Agricultural Research Services (USDA-ARS) facilities in Hawaii by McInnis et al. (2004) for sterile male releases in Mauritius would have been at risk. Mating compatibility studies between populations from Mauritius, Seychelles and the genetic sexing strain from Hawaii using the Mating Performance Field Cage Test showed, however, that the flies were fully compatible with no evidence for incipient speciation in spite of the differences in host use (Sookar et al. 2013).

*Anastrepha ludens* (Loew) is a species that belongs to the *fraterculus* group and is a major pest in Mexico and other Central American countries (Aluja 1994). A mass-rearing facility in southern Mexico provides sterile flies to different regions of Mexico. Orozco-Dávila et al. (2007) using the same Mating Performance Field Cage Test evaluated the compatibility between the mass-reared strain and six wild populations from these regions and found that this strain was fully compatible with all the wild populations tested and that the sterile males were also competitive with wild males. However, some subtle differences in the time of male calling activity were detected in the wild populations with some calling earlier than others; differences that could be the result of adaptations to the local environments in different latitudes as seen for the case of *Bactrocera* (Schutze et al. 2013). Studies including both *Anastrepha ludens* and *Anastrepha obliqua* (Macquart) on a field-caged mango tree (larvae of both species can be found infesting mango) showed distinct temporal pattern of sexual activities for each species, although there was a small overlap of male calling activities, during which the formation of mixed leks was observed (Aluja et al. 1983).

*Anastrepha fraterculus* is a species with a wide geographical range (Steck 1999) and high levels of variability in morphology, cytology and genetics, suggesting that it consists of a cryptic species complex (Stone 1942, but see Steck 1999 for a review) that includes different species (Selivon 1996, Yamada and Selivon 2001, Selivon et al. 2004) or morphotypes (Hernández et al. 2004, 2012, 2015). In order to assess whether this variability is accompanied with mating incompatibility among different populations, studies involving several morphotypes and geographical scales were performed using the Mating Performance Field Cage Test (Table 1). Petit-Marty et al. (2004a) evaluated the mating compatibility among four populations from Argentina, two from the northwest and two from the northeast of the country. All pair-wise mating combinations were compatible and additional studies on post-zygotic barriers confirmed full compatibility among populations (Petit-Marty et al. 2004b). The genetic (Alberti et al. 2002) and morphometric (Hernández-Ortiz et al. 2012) characterization of these populations confirmed that they all belonged to the Brazilian-1 morphotype (Hernández-Ortiz et al. 2012). The study of Petit-Marty et al. (2004a) was extended on a regional scale by Vera et al. (2006) who evaluated the mating compatibility among six populations of *Anastrepha fraterculus* from three different morphotypes (Brazilian-1, Peruvian and Andean). These authors found all levels of mating isolation among different populations, ranging from full compatibility to high incompatibility. Within morphotypes, the results ranged from full compatibility to moderate incompatibility (as in the case of Tucumán from Argentina and Piracicaba from Brazil, Table 1). However, among different morphotypes, mating isolation was always significant. The study also revealed the occurrence of temporal differences in sexual activity patterns of some of the populations: while the populations from the Brazilian-1 morphotype mated early in the morning, the Peruvian ones mated at midday and the Andean ones mated in the evening. Interestingly, the cases of moderate isolation observed within some morphotypes were accompanied by differences in the location of the mated couples. More recently, Rull et al. (2012) evaluated the compatibility of three strains belonging to the Brazilian-1 morphotype, i.e. two laboratory strains from southern Brazil and one strain from Argentina, and found them fully compatible. This result was later confirmed by a study with wild flies covering five populations from south and southeast Brazil that also belonged to the Brazilian-1 morphotype (Dias 2012). On the other hand, the evaluation of the compatibility between populations from the Mexican morphotype and those from other morphotypes (Peruvian and Brazilian-1) showed strong incompatibility with no difference of the time of sexual activity when compared to the Brazilian-1 morphotype (Rull et al. 2013). Devescovi et al. (2014) expanded the evaluation of the Andean morphotype, confirmed the mating isolation with populations of the Peruvian morphotype and extended it to those of the Brazilian-1 morphotype. Two recent reviews of this subject can be found in Cladera et al. (2014) and Vaníčková et al. (2015a). Cladera et al. (2014) discuss the implications of mating compatibility for SIT application against *Anastrepha fraterculus*, while Vaníčková et al. (2015a) discuss the data obtained from mating incompatibility among Brazilian populations in a broader perspective, including sexual behavior, post-zygotic studies, and chemical ecology.

**Table 1.** Summary of sexual isolation indices from field cage tests carried out for the *Anastrepha fraterculus* complex.

Reference	Population – mating combination	Morphotypes combination	ISI	Isolation level
**Petit-Marty et al. 2004**	Tucumán (Arg) – Entre Ríos (Arg)	Brazilian-1 – Brazilian-1	–0.01 ± 0.17	Random mating
	Tucumán (Arg) – Misiones (Arg)	Brazilian-1 – Brazilian-1	–0.03 ± 0.05	Random mating
	Jujuy (Arg) – Tucumán (Arg)	Brazilian-1 – Brazilian-1	– 0.01 ± 0.05	Random mating
	Jujuy (Arg) – Entre Ríos (Arg)	Brazilian-1 – Brazilian-1	– 0.04 ± 0.17	Random mating
	Jujuy (Arg) – Misiones (Arg)	Brazilian-1 – Brazilian-1	–0.09 ± 0.09	Random mating
	Misiones (Arg) – Entre Ríos (Arg)	Brazilian-1 – Brazilian-1	–0.09 ± 0.13	Random mating
**Vera et al. 2006**	La Molina (Peru) – Entre Ríos (Arg)	Peruvian – Brazilian-1	0.92 ± 0.03	High
	Tucumán (Arg) – Piura + La Molina (Peru)	Brazilian-1 – Peruvian	0.83 ± 0.06	High
	Tucumán (Arg) – La Molina (Peru)	Brazilian-1 – Peruvian	0.82 ± 0.03	High
	La Molina (Peru) – Ibague (Col)	Peruvian – Andean	0.78 ± 0.02	High
	La Molina (Peru) – Piracicaba (Br)	Peruvian – Brazilian-1	0.55 ± 0.06	Moderate
	Tucumán (Arg) – Piracicaba (Br)	Brazilian-1 – Brazilian-1	0.43 ± 0.08	Moderate
	Tucumán (Arg) – Entre Ríos (Arg)	Brazilian-1 – Brazilian-1	0.12 ± 0.10	Random mating
	La Molina (Peru) – Piura + La Molina (Peru)	Peruvian – Peruvian	0.10 ± 0.12	Random mating
**Cáceres et al. 2009**	Tucumán (Arg) – La Molina (Peru)	Brazilian-1 – Peruvian	0.77 ± 0.05	High
	Tucumán (Arg) – La Molina (Peru)_Unisex Arg_	Brazilian-1 – Peruvian	0.73 ± 0.05	High
	Tucumán (Arg) – La Molina (Peru)_Unisex Peru_	Brazilian-1 – Peruvian	0.86 ± 0.04	High
	Hybrid ArgPeru – Arg_UnisexArg_	Hybrid Brazilian-1 /Peruvian – Brazilian-1	0.30 ± 0.12	Moderate
	Hybrid PeruArg – Arg_UnisexArg_	Hybrid Peruvian /Brazilian-1 – Brazilian-1	0.15 ± 0.11	Random mating
	Hybrid ArgPeru – Peru_UnisexPeru_	Hybrid Brazilian-1 /Peruvian – Peruvian	0.10 ± 0.10	Random mating
	Hybrid PeruArg – Peru_UnisexPeru_	Hybrid Peruvian /Brazilian-1 – Peruvian	0.13 ± 0.09	Random mating
**Rull et al. 2012**	Tucumán (Arg) – Vacaria (Br)	Brazilian-1 – Brazilian-1	0.12 ± 0.06	Random mating
	Tucumán (Arg) – Pelotas (Br)	Brazilian-1 – Brazilian-1	0.14 ± 0.09	Random mating
	Pelotas (Br) – Vacaria (Br)	Brazilian-1 – Brazilian-1	0.14 ± 0.08	Random mating
**Dias 2012**	Pelotas (Br) – Bento Gonçalves (Br)	Brazilian-1 – Brazilian-1	0. 14 ± 0.07	Random mating
	São Joaquim (Br) – Vacaria (Br)	Brazilian-1 – Brazilian-1	0.04 ± 0.04	Random mating
	São Joaquim (Br) – Bento Gonçalves (Br)	Brazilian-1 – Brazilian-1	0.14 ± 0.07	Random mating
	Bento Gonçalves (Br) – Vacaria (Br)	Brazilian-1 – Brazilian-1	0.03 ± 0.05	Random mating
	Piracicaba (Br) – São Joaquim (Br)	Brazilian-1 – Brazilian-1	0.55 ± 0.09	Moderate
	Piracicaba (Br) – Bento Gonçalves (Br)	Brazilian-1 – Brazilian-1	0.56 ± 0.05	Moderate
	Piracicaba (Br) – Vacaria (Br)	Brazilian-1 – Brazilian-1	0.53 ± 0.10	Moderate
**Rull et al. 2013**	Xalapa (Mex) – Tucumán (Arg)	Mexican – Brazilian-1	0.82 ± 0.06	High
	Xalapa (Mex) – Vacaria + Pelotas (Br)	Mexican – Brazilian-1	0.89 ± 0.02	High
	Xalapa (Mex) – La Molina (Peru)	Mexican – Peruvian	0.74 ± 0.03	High
Devescovi et al. 2014*	Tucumán (Arg) – Ibague (Col)	Brazilian-1 – Andean	1	High
	Xalapa (Mex) – Ibague (Col)	Mexican – Andean	0.94	High
	La Molina (Peru) – Ibague (Col)	Peruvian – Andean	0.65	Moderate-High

* ISI values were estimated from Table 1.

## Are results from walk-in field cage tests reliable?

There is no doubt that mating compatibility studies carried out in small cages under laboratory conditions can result, because of the high densities and close proximity of flies due to the limited space, in interspecific matings that normally would not occur under natural or even semi-natural conditions. The unreliability of small cage mating tests and the need to develop a field cage method that reflects more the natural situation was realized at the early stages of fruit fly SIT programs (Boller 1977, Prokopy and Hendrichs 1979, Aluja et al. 1983). In addition, Japanese *Zeugodacus cucurbitae* researchers found that the mating competitiveness of mass-reared vs. wild melon flies varied depending on the size of laboratory cages and fly density. As size increased, the competitiveness of wild flies increased, whereas that of the laboratory-reared flies decreased, confirming the need to carry out these assessments under walk-in field cage conditions to eliminate this distortion (Koyama 1982, Kuba et al. 1984).

Since then, the field cage test that is routinely used in SIT programs around the world has gradually evolved (FAO/IAEA/USDA 2014). After several decades of experience with the Mating Performance Field Cage Test a wealth of information confirmed its reliability. Despite this wealth of data, some still might question the usefulness of walk-in field cages as reliable tools to evaluate mating compatibility within the Tephritidae. Nevertheless, all evidence points towards a positive answer with the most convincing arguments being that a) under the same experimental conditions, using a field-caged host tree, an entire range of outcomes can be obtained: from full mating compatibility among some populations to complete assortative mating among others, b) these outcomes can be replicated by different research teams, and c) they have been fully endorsed from evidence obtained following simultaneously molecular, morphological and other approaches. The capacity of the field cage test to measure the degree of sexual compatibility was again shown in these recent studies described above, involving the *Anastrepha fraterculus* cryptic species and *Bactrocera dorsalis* species complexes, helping to define species limits and even leading to synonymization in the case of the *Bactrocera dorsalis* complex (Schutze et al. 2015a).

As a standard index, the Index of Sexual Isolation (ISI, see Box 1) has been found adequate to provide a measure of the level of mating isolation between populations (Table 1). Based upon the accumulated data, the following scale is proposed: ISI between -0.2 and 0.2 for random mating (or higher range provided that the 95% confidence interval includes zero), ISI between 0.2 and 0.5 for moderate yet statistically significant isolation (when the 95% confidence interval does not include zero), ISI between 0.5 and 0.7 for moderate to high isolation and ISI above 0.7 for strong isolation (Table 1). In addition, other relevant measures, such as, Relative Isolation Index (RII), Male Relative Performance Index (MRPI), Female Relative Performance Index (FRPI) (see Box 1), mating location and latency to mate also contribute to understand the mechanisms involved in mating isolation. For instance, the RII was shown to be more sensitive to slight changes in the number of mating pairs obtained from the different mating combinations (FAO/IAEA/USDA 2014); MRPI and FRPI provide information on the readiness of each sex from the two populations to mate and this has proven to be a reliable indicator of how populations and strains can be more or less competitive (males) or receptive (females); a different mating location of the mating pairs has been explained by the occurrence of spatial isolation; and differences in the timing of sexual activity of the male and female flies has reflected temporal isolation.

### How to further refine and obtain additional information from field cage tests?

Although mating compatibility studies in walk-in field cages have been used to delimit species boundaries, there are variants to the standard protocol that can contribute to complete the picture of the potential acting isolation mechanisms. Here we discuss some of these possible variants and provide examples in which these modifications were found to be beneficial.

### Changing the sex ratio or replacing flies

The general recommendation for the Mating Performance Field Cage Test is to release the flies at a 1:1 male:female ratio. However, this can bias measures of pre-zygotic compatibility for populations which show subtle differences in the timing of mating. For instance, the already mentioned study of Schutze et al. (2013) revealed slight differences in the time of sexual activity of the populations and species under study. Therefore the authors were concerned about the possibility that males and females of the population that engaged in earlier matings deprived males of the population that engaged in sexual activities later from a potential mating with a heterotypic female (as these would have been removed already as part of the procedure to extract formed mating pairs from the field cage, see Box 1), even in the absence of sexual incompatibility. Therefore, the authors proposed a protocol to use a 1:2 male:female ratio in the field cage to ensure an excess of females allowing heterotypic crosses independent of timing of mating. Alternatively, mating pairs can be replaced with virgin flies of the same population as they form. This way, the cage always would contain the same number of males and females of each population or origin, independent of the number of mating pairs that formed and have been removed. However, the latter mentioned protocol may disturb the flies in the field cage and newly released males will lack the acclimatization period. Therefore, our recommendation is to keep the male:female ratio at 1:1 and only if differences in time of mating are revealed, then consider the possibility of changing the sex ratio.

### Switching the time of sexual activity

One alternative to avoid the effect of a different timing of sexual activity on mating compatibility is to attempt matching the mating time of flies from different populations. This can potentially be achieved by maintaining the flies during their pre-copulatory period, which depending on the species can vary between 2–30 days, under different light regimes to synchronize the peaks of their mating times. This approach was used by Vera et al. (2006) when they evaluated an *Anastrepha fraterculus* population from Peru and one from Argentina, which showed mating incompatibility due to differences in their timing of sexual activity. While Argentinean flies mated early in the morning (mean latency to mate < 1h), Peruvian flies became sexually active 3–4 h later. To synchronize their sexual activity periods, Peruvian flies were held after emergence for two weeks in a room in which the lights were turned on 4 h earlier than in the room that contained the Argentinean flies. Flies were released in the field cages at sunrise under natural light conditions. Results showed that although Peruvian flies advanced their mating peak period by approximately one hour, and hence, the overlap of the timing of sexual activity between the two populations increased, the number of heterotypic matings remained low, confirming the presence of strong sexual isolation. Further research with other morphotypes and species complexes is needed to confirm whether this approach can be more widely applied to dissect and better understand mating incompatibility.

### What material to select for the evaluations?

In many cases, sourcing of the flies can be problematic as often populations, morphotypes, or species can be hard to find in nature or for which collection, transport, export or import permits are difficult to obtain. In those cases, compatibility studies require the establishment of laboratory colonies and this raises concerns on the extent, degree and impact of laboratory adaptation on the sexual behavior of the flies. Reduction of male competitiveness, changes in male courtship, and increase in female receptivity associated to mass-rearing are frequently documented in the literature (Iwahashi 1996, McInnis et al. 1996, Briceño and Eberhard 1998, Rull et al. 2005). To avoid this, the use of wild flies or strains only recently introduced into the laboratory and maintained using natural fruit as oviposition and larval rearing substrate under relaxed conditions (i.e. “wildish” strains) is recommended.

The wide range of mating compatibility studies performed over the last years has generated data that allow assessing the impact of laboratory rearing on mating compatibility. Two populations of *Bactrocera dorsalis* originating from central and southern Thailand showed initially significant positive assortative mating as a result of differences in latency to mate and the degree of male and female fly participation in mating. After one year of rearing under the same laboratory conditions, flies from the same populations mated at random and no differences in latency or female receptivity were detected (Schutze et al. 2015b). The change from positive assortative to random mating was attributed by the authors to changes in mating latency and relative participation of the sexes in mating. The change in mating time was associated with adapting the populations originating from different latitudes to the same local environmental conditions, whereas the reasons for differential mating participation remain to be resolved. The authors concluded that for the purpose of species delimitation, taking alone the results of the wildish colonies would have resulted in wrong conclusions. As such, they recommended the use of laboratory cultures after the initial adaptation to the same laboratory conditions but not exceeding the sixth generation. Different results however, were obtained with populations of *Anastrepha fraterculus*. After three years of identical laboratory rearing at the same facility, populations from two different morphotypes remained isolated even though populations originated from different latitudes (Cáceres et al. 2009). Differences in the timing of mating and in the chemical profiles of male-borne volatiles were, among others, possible explanations for this mating isolation. Taken together, results showed that subtle differences in mating time of the *Bactrocera dorsalis* populations probably originated from some kind of local adaptation of the flies to particular environmental conditions in the area of origin, can be removed under identical rearing conditions, while for the case of *Anastrepha fraterculus* the initial isolation barriers between the two morphotypes remained even after a longer period of identical rearing conditions. It could be argued in the *Bactrocera dorsalis* case, that the differences were not strong enough to keep the populations isolated when maintained under similar environmental conditions, while in the *Anastrepha fraterculus* case they were strong enough to persist. Since it is always very difficult to know in advance the strength of isolating mechanisms, it is safer to use wild flies or populations recently introduced into the laboratory. We concur also with the recommendation of Schutze et al. (2015b) to use recently colonized populations that have been given some time to adapt to similar environmental conditions when subtle adaptations to the environment are expected. However, one should not neglect colonies that have been cultured for a longer time if wild material is scarce or difficult to obtain.

### Releasing females from one origin only

Wee and Tan (2000) used a variant of the Mating Performance Field Cage Test in which females from one population were released rather than females from two populations. This approach was evaluated with *Bactrocera papayae* and *Bactrocera carambolae* and the data indicated that *Bactrocera papayae* females were selective and preferred to mate with con-specific males, while *Bactrocera carambolae* females did not discriminate between males of the two populations. The same no-choice approach was used by Cáceres et al. (2009) with *Anastrepha fraterculus* populations from Peru and Argentina. High ISI values were obtained both when females from two origins were used and when females from one origin were used. Although releasing both sexes is closer to the natural situation, the use of females from one population may be justified in well-defined cases when they do not seem to bias the results obtained. This variant avoids also that females from one population sequester the males from their own origin. On the other hand, using only wild females of the target population is closer to the situation when mass-rearing and releasing sterile males of a genetic sexing strain in an SIT program. Therefore when assessing the competitiveness of males of genetic sexing strains, the preferred option is to have laboratory males (non-irradiated or irradiated) compete with wild males from a target population in the absence of laboratory females.

### Evaluating hybrids

Hybrids derived from populations or morphotypes that show sexual isolation under walk-in field cage conditions, but that can produce progeny under high-density conditions using small laboratory cages, can be a fertile ground to understand speciation processes. For example, male hybrids obtained under such conditions from matings between *Anastrepha fraterculus* populations from Peru and Argentina produced a pheromone that was a mix of the parental pheromones (Cáceres et al. 2009), and the hybrid females preferred mating with hybrid males rather than with males of the parental populations (Segura et al. 2011). Interestingly, parental females did not discriminate between the males of their own morphotype and the hybrid males (Cáceres et al. 2009), probably indicating that parental females from both morphotypes use the compounds present in the pheromones of their con-specific males to recognize them, even when hybrid males’ pheromone had different proportions of these compounds and, what is more, the presence of additional compounds that are not part of the parental pheromone. The marked preference of hybrid females for hybrid males induced Segura et al. (2011) to postulate that hybrid speciation has the potential to occur in nature and could be one of the reasons for the high number of taxonomic entities that comprise this *Anastrepha fraterculus* cryptic species complex. In addition, it showed the probable significance of chemical cues for species recognition within the *Anastrepha fraterculus* cryptic species complex, highlighting the need for specific research on this topic.

## The role of pheromones in species recognition

The ability to find a mate depends on the recognition between members of the same species. The sensory system is a significant component of sexual communication across many insect taxa and plays an important role in pre-mating isolation (Symonds and Elgar 2008, Smadja and Butlin 2009). These sensory systems consist of sensory neuronal receptors and neuronal pathways that allow discrimination of individual sensory modalities and their mutual integration. In many species, visual, acoustic, tactile and chemical signals are all involved in the pre-mating communication process. If stabilizing selection acts on the sexual communication system, any change made can lead to a process of evolutionary divergence which may be the initial step towards behavioral isolation within a process of speciation (Symonds and Elgar 2008, Smadja and Butlin 2009). Consequently, understanding the role of each signal on the evolution of reproductive barriers is likely to be important for understanding speciation.

### *Anastrepha fraterculus* mating system and chemical communication

As in many others, but not all, tephritid flies, *Anastrepha fraterculus* has a lek mating system (Malavasi et al. 1983) in which males aggregate and emit different visual, acoustical and chemical signals to attract females to a courting arena (a behavior known as “calling”). Once in the lek, the female fly assesses the multimodal calling behavior of a number of calling males before selecting a mate among the available ones. There is no data available about what modality is crucial in female choice. In general, chemical signals are supposed to play an important role both in female attraction and female choice. Long range (pheromones) and close range (cuticular hydrocarbons) signals are involved. Pheromones are produced in salivary and anal glands (Nation 1989) and are released by expanding the lateral pouches of the pleural abdominal cuticle, and the periodic protrusion of anal tissue which forms a pouch. Usually, this behavior is accompanied by rapid wing movements (fanning) that produce vibrations and a flux of air over the body surface, thought to enhance the diffusion of pheromone (Nation 1989, Segura et al. 2007, Lima-Mendonça et al. 2014, Bachmann et al. 2015). Although the exact factors of mate selection by the females are not fully understood, male copulatory success seems to be related, at least in part, to pheromone calling activity (Bachmann et al. 2015), the male location within the lek (Segura et al. 2007), and specific morphological traits such as wing width and thorax length (Sciurano et al. 2007) as well as eye length (Segura et al. 2007). Female mating preference seems therefore to be the result of the integration of multiple stimuli. Although there are no formal recordings, observations in walk-in field cages revealed that males belonging to different morphotypes called in the same tree or even formed mixed leks (for *Anastrepha ludens* and *Anastrepha obliqua* see also Aluja et al. 1983) from which males courted females from the other morphotypes, suggesting that species recognition occurs during courtship and probably the females play a key role at the time of mate selection to avoid cross-mating. As such *Anastrepha fraterculus* females possibly use all the signals emitted by the males during courtship for species recognition, especially those that present differences among morphotypes.

The volatiles emitted by the males seem to vary both in quality and quantity across morphotypes within the *Anastrepha fraterculus* complex (Cáceres et al. 2009, Břízová et al. 2013, Vaníčková et al. 2015b, Table 2), and appear to be playing a role in more long-range interactions and courtship, while the less volatile signals and cuticular hydrocarbons mediate close-range chemical interactions (Vaníčková et al. 2015b). In addition, females from the different morphotypes showed differences in their antennal response (Břízová et al. 2013, Milet-Pinheiro et al. 2014), i.e. while the antenna of Tucumán, Argentina (Brazilian-1 morphotype) females responded to (*Z*, *E*)-a-Farnesene, (*E*, *E*)-a-Farnesene, Epianastrephin, (*Z*)-3-Nonen-1-ol and (*E*, *Z*)-3,6-Nonadien-1-ol (Břízová et al. 2013), the antenna of Alagoas (Brazilian-3 morphotype) females responded to a- Pinene, Limonene, (*Z*)-3-Nonen-1-ol and (*E*, *Z*)-3,6-Nonadien-1-ol, (S,S)-(-)-Epianastrephin (Milet-Pinheiro et al. 2014). Although it has been shown that these compounds attracted females of the Brazilian-3 morphotype and that the females responded equally to an artificial blend of the synthetic compounds and to volatiles collected from males, it is still not known whether these differences observed at the chemical and electrophysiological level are translated into different behaviors, since the behavioral response to the pheromone of the Argentinean populations is not known yet. Therefore, in order to elucidate this, it is necessary to determine the degree of intra- and inter-specific variability in males’ pheromone and in female’ pheromone perception and especially to conduct behavioral tests in which females are faced with the pheromones of con-specific and hetero-specific males simultaneously to determine the role of pheromone signaling for species recognition. The same would be interesting to perform with cuticular hydrocarbons that are supposed to mediate the close-range chemical recognition.

**Table 2.** Chemical profiles of the male-borne volatiles from different populations from the *Anastrepha fraterculus* complex.

	Morphotype										
Compound		Brazilian-1									
		Tucumán	Tucumán	Bento Gonçalves	Bento Gonçalves	Pelotas	Pelotas	São Joaquim	São Joaquim	Piracicaba	Vacaria
	RI	1	2	2	3	2	3	2	3	2	2
3-Hexanone	791	wi	wi	wi	wi	wi	wi	wi	wi	wi	wi
2-Hexanone	796	wi	wi	wi	wi	wi	wi	wi	wi	wi	wi
Hexanal	801	wi	wi	wi	wi	wi	wi	wi	wi	wi	wi
α-Pinene	938	wi	wi	wi	+	wi	++	wi	++	wi	wi
Camphene	956	wi	wi	wi	wi	wi	wi	wi	wi	wi	wi
β-Pinene	985	wi	wi	wi	wi	wi	wi	wi	wi	wi	wi
Myrcene	991	wi	wi	wi	wi	wi	wi	wi	wi	wi	wi
Ethyl hexanoate	996	wi	wi	wi	wi	wi	wi	wi	wi	wi	wi
*p*-Cymene	1022/1030	wi	+	+	wi	+++	wi	+	wi	+	+
2-Ethylhexan-1-ol	1029/1030	wi	++	++	wi	+	wi	+	wi	+	+++
Limonene*^2^	1041/1035	-	++	++	+++	+++	+++	+++	+++	+	++
5-Ethenyldihydro-5-methyl-2(3H)-furanone	1044	wi	wi	wi	wi	wi	wi	wi	wi	wi	wi
Indane	1046	wi	wi	wi	wi	wi	wi	wi	wi	wi	wi
(Z)-β-Ocimene	1050/1035	wi	++	+	wi	+	wi	+	wi	++	tr
(E)-β-Ocimene	1059	+	wi	wi	wi	wi	wi	wi	wi	wi	wi
Linalool	1101	wi	wi	wi	wi	wi	wi	wi	wi	wi	wi
(Z)-Nonanal	1107	+	tr	++	wi	+	wi	+	wi	+	+++
Camphor	1141	wi	wi	wi	wi	wi	wi	wi	wi	wi	wi
(Z)-3-Nonen-1-ol*^12^	1159/1158	+++	+	++	++	tr	++	+	++	+	+
(E,Z)-3,6-Nonadien-1-ol*^12^	1161/1160	wi	++	+++	+++	tr	++	++	+++	+++	+
Decenal	1210	wi	tr	+	wi	+	wi	+	wi	+	+
Bornyl acetate	1293	wi	wi	wi	wi	wi	wi	wi	wi	wi	wi
(E)-α-Bergamontene	1435	-	wi	wi	wi	wi	wi	wi	wi	wi	wi
(Z)-β-Farnesene	1448	wi	wi	wi	wi	wi	wi	wi	wi	wi	wi
(Z,E)-α-Farnesene*^12^	1495/1492	+++	+	+	+	+	—	+	+	+	+
Germacrene D	1498/1502	wi	tr	+	wi	+	wi	+	wi	+	+
Suspensolide	1506/1509	+++	+	++	wi	+	wi	+	wi	++	+
(E,E)-α-Farnesene*^12^	1512/1510	+++	+++	++	+++	++	+++	+++	+++	+++	+
Anastrephin	1617/1610	+++	+	+	wi	+	wi	+	wi	+	+
Caryophyllene oxide	1606	wi	wi	wi	wi	wi	wi	wi	wi	wi	wi
Epianastrephin*12	1621/1625	+++	+	+	+	+	+++	++	+	+	+
Benzoic acid	wi	+++	wi	wi	wi	wi	wi	wi	wi	wi	wi
β-Bisaboline	wi	-	wi	wi	wi	wi	wi	wi	wi	wi	wi

	Morphotype								
Compound		Brazilian-3			Peruvian	Andean			
		Alagoas	Alagoas	Alagoas	La Molina	Ibague	Sibundoy	Duitama	Cachipay
	RI	2	4	3	1	3	3	3	3
3-Hexanone	791	wi	tr	wi	wi	wi	wi	wi	wi
2-Hexanone	796	wi	+	wi	wi	wi	wi	wi	wi
Hexanal	801	wi	+	wi	wi	wi	wi	wi	wi
α-Pinene	938	wi	+	+	wi	+++	+++	+++	+++
Camphene	956	wi	tr	wi	wi	wi	wi	wi	wi
β-Pinene	985	wi	+	wi	wi	wi	wi	wi	wi
Myrcene	991	wi	+	wi	wi	wi	wi	wi	wi
Ethyl hexanoate	996	wi	+	wi	wi	wi	wi	wi	wi
*p*-Cymene	1022/1030	++	+	wi	wi	wi	wi	wi	wi
2-Ethylhexan-1-ol	1029/1030	tr	+	wi	wi	wi	wi	wi	wi
Limonene*^2^	1041/1035	+++	+++	+++	+	+++	+++	+++	+++
5-Ethenyldihydro-5-methyl-2(3H)-furanone	1044	wi	+	wi	wi	wi	wi	wi	wi
Indane	1046	wi	+	wi	wi	wi	wi	wi	wi
(Z)-β-Ocimene	1050/1035	—	+	wi	wi	wi	wi	wi	wi
(E)-β-Ocimene	1059	wi	+	wi	+++	wi	wi	wi	wi
Linalool	1101	wi	tr	wi	wi	wi	wi	wi	wi
(Z)-Nonanal	1107	+	wi	wi	+	wi	wi	wi	wi
Camphor	1141	wi	tr	wi	wi	wi	wi	wi	wi
(Z)-3-Nonen-1-ol*^12^	1159/1158	+	tr	++	+	+	+	+	+
(E,Z)-3,6-Nonadien-1-ol*^12^	1161/1160	+++	+	+++	wi	+	+	+	+
Decenal	1210	+	wi	wi	wi	wi	wi	wi	wi
Bornyl acetate	1293	wi	+	wi	wi	wi	wi	wi	wi
(E)-α-Bergamontene	1435	wi	+	wi	+++	wi	wi	wi	wi
(Z)-β-Farnesene	1448	wi	+	wi	wi	wi	wi	wi	wi
(Z,E)-α-Farnesene*^12^	1495/1492	+	+	+	-	+	+	+	+
Germacrene D	1498/1502	+	tr	wi	wi	wi	wi	wi	wi
Suspensolide	1506/1509	++	+	wi	+++	wi	wi	wi	wi
(E,E)-α-Farnesene*^12^	1512/1510	+	+++	—	+++	+	+	+	+
Anastrephin	1617/1610	+	+	wi	+++	wi	wi	wi	wi
Caryophyllene oxide	1606	wi	tr	wi	wi	wi	wi	wi	wi
Epianastrephin*12	1621/1625	+	+	+	+++	+	+	+	+
Benzoic acid	wi	wi	wi	wi	-	wi	wi	wi	wi
β-Bisaboline	wi	wi	wi	wi	+++	wi	wi	wi	wi

1 – Cáceres et al. 2009; 2 – Břízová et al. 2013; 3 – Vaníčková et al. 2015; 4 – Milet-Pinheiro et al. 2014 *1 2 = Břízová et al. 2013 + Milet-Pinheiro et al. 2014 – antennally active; *2 = Milet-Pinheiro et al 2014 – antennally active +++ = large amounts (³ 19%); ++ = medium amounts (7–19%); + = small amounts (1–7%); tr = traces (< 0.1%); - = no detectable amounts; wi = without information

### The response of *Anastrepha fraterculus* females to calling males

Walk-in field cages can also be useful to explore the role of chemical communication in mate finding and species recognition and the Manual for Product Quality Control for Sterile Mass-Reared and Released Tephritid Fruit Flies (FAO/IAEA/USDA 2014) provides a protocol to carry out pheromone attraction tests in field cages. This protocol has the advantage that it can provide information on both relatively long and close distance recognition. Several examples of the use of walk-in field cages to assess tephritid attraction to different odor sources can be found in the literature (Webb et al. 1983, Shelly 2000a, 2000b, López-Guillén et al. 2011, Liendo et al. 2013, Milet-Pinheiro et al. 2014). However, the usefulness of walk-in field cages to assess preference of female flies for pheromones of con-specific vs hetero-specific males has not been evaluated yet.

To determine whether *Anastrepha fraterculus* male pheromones from two different populations were equally attractive to con-specific and hetero-specific females, the methodology of Liendo et al. (2013) was adapted, which in turn is an adaptation of the methodology proposed by Shelly (2000a) for *Ceratitis capitata* (Box 2). The experiments were carried out in a greenhouse of the FAO/IAEA Insect Pest Control Laboratory (IPCL) in Seibersdorf, Austria and involved one population from Piracicaba, São Paulo, Brazil and one from Tucumán, Argentina. Obtained results show that orientation and location of the female flies in the field cages was affected by the presence of calling males (Table 3). In the control cages in which only one type of male was calling (the other containers were empty controls), it was found that Piracicaba females preferred the tree and the leks with calling males instead of those with no males. In a similar way, Tucumán females preferred the artificial leks with males instead of the empty containers. In the cages in which males of the two populations were calling, females showed no particular preference; leks of the two origins were visited at equal rates. This is in agreement with the fact that these populations have been both identified as belonging to the Brazilian-1 morphotype. However, considering previous chemical and behavioral data, some differences could have been expected. Quantitative differences were found in the pheromone compounds of these populations. (Břízová et al. 2013, Table 2). In addition, some mild, yet significant, mating isolation associated with some spatial distribution within the field cage (homotypic couples were found in different areas of the cage) was found between Tucumán and Piracicaba (Vera et al. 2006) and between Piracicaba and other Brazilian-1 morphotype populations from Southern Brazil (Dias 2012). It can be argued that these differences could have affected the frequency of female visits to a lek; however our results suggest that mate recognition may have occurred later, when the females were already in closer vicinity of the males and other signals (visual, vibratory or chemical related to differences in the cuticular hydrocarbons [Vaníčková et al. 2012]) might have been important.

**Box 2.** Female orientation to male pheromone.

**Figure 1.**
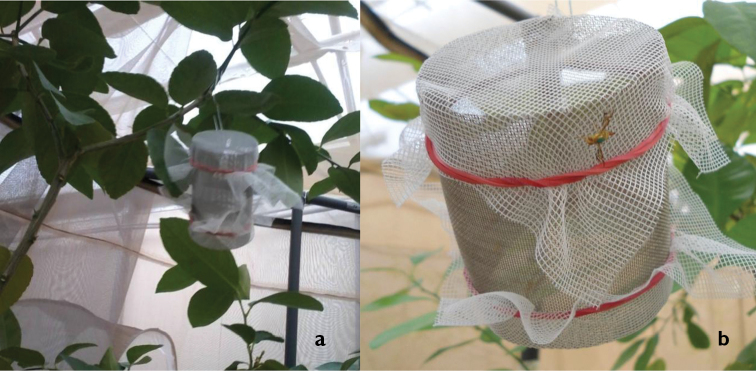
Walk-in field cage set up to evaluate female response to male pheromone: **a** artificial lek hanging from the tree **b** *Anastrepha fraterculus* female over an artificial lek.

For the purpose of evaluating the response of females towards the male pheromone, an indicator of intra-specific recognition in lek-forming tephritids, field cages are set up with two potted trees inside, which are virtually divided into two sectors. Each sector contains one tree. The test involves two steps. In the first, it is determined whether females orient to the pheromone of con-specific males and 25 mature virgin females of a given population are released into the field cage during the period of sexual activity. Fifteen minutes later, 3 “artificial leks” consisting of cylindrical metal wire-mesh containers (3 cm diam., 7 cm long) with 7 sexually mature males inside (Figure 1) are hung in one of the trees while 3 containers without males are hung in the other tree. Once the females and the containers are placed in the cage, an observer scores the number of females in each sector, in each tree and those on the artificial lek. These parameters are recorded every 15–20 minutes, with at least six observations made during the period of mating activity in each cage. If significant differences in preference towards male-containing leks are found (see below), it is possible to continue with the second step, which involves the evaluation of the ability of the females to distinguish between male pheromones of different populations, morphotypes or species. To do so, 25 mature virgin females from two different populations are released inside each field cage, into which artificial leks with males from the same two populations are hung from the trees. Each tree houses 3 containers from one population. In order to identify females from each origin, the day before the test flies are painted with a dot of water based acrylic in their thorax. Colors are randomly assigned and permuted every day. Observations are performed as described previously. To prevent pheromone contamination, trees and cages are washed with pressurized tap water at the end of each test. The containers used for the artificial leks are also washed with hot water and dried in an oven at 150 °C for one hour. To avoid females behaving differently due to adaptation of sensory organs and due to habituation in the brain, males and females should be kept in separate rooms so that females have no previous experience with the male pheromones. Data from the daily observations within each cage (i.e. replicate) are added to obtain an overall measure of the location of the females throughout the experiment. Statistical analysis is done by means of a paired t-test or the corresponding non-parametric Wilcoxon paired test as appropriate, in which the number of flies registered in the area, in the tree and at the artificial lek is compared between sectors. In the first step (i.e. field cages with empty control containers), significant differences indicate that females use chemical cues to find males. In the second step (i.e. comparing male types), significant differences indicate that females orient towards one male type, while non-significant differences indicate either lack of capacity to discriminate or no preference towards any male type.

**Table 3.** Results from the Wilcoxon sign rank test to evaluate orientation *Anastrepha fraterculus* females from different populations to artificial leks (containers with sexually mature males).

			Area		Tree		Lek	
Lek combination	Female	N	Z	p-level	Z	p-level	Z	p-level
Control^†^	Piracicaba	5	0,73	0,4652	2,02	0,0431	2,02	0,0431
Control	Tucumán	8	1,12	0,2626	1,12	0,2626	2,20	0,0277
Tucumán – Piracicaba	Piracicaba	7	0,54	0,5896	1,35	0,1763	0,40	0,6858
Tucumán – Piracicaba	Tucumán	7	1,86	0,0630	0,51	0,6121	0,53	0,5930

† In the control cages one of the two trees had empty containers with no males inside.

The results presented above showed that walk-in field cages can be used to measure the response of *Anastrepha fraterculus* females towards volatiles emitted by *Anastrepha fraterculus* calling males and this opens opportunities to better understand the mechanisms behind mating isolation between morphotypes. The experimental protocol, however, entails two issues that need to be resolved: first, the comparisons are restricted to populations that have the same timing of sexual activity; second, it is not possible to control the amount of chemical stimuli released as the number of males that are calling at any particular time within the artificial lek cannot be controlled. The former can be solved by changing the photoperiod of those populations that have different time of sexual activity, while for the latter it is advisable to monitor the number of males calling during the test, as for this species, the numbers of males calling was correlated with the amount of pheromone released (Bachmann et al. 2015). Along with the monitoring of calling males, several mating cages can be operated in parallel, in which males and females are released at the same time of the pheromone attraction test to confirm female readiness to mate.

An alternative approach to solve differences in calling times and in number of calling males is the use of collected volatiles or artificial blends made of synthetics pheromone analogs in the right proportions. Such an approach has been evaluated by Milet-Pinheiro et al. (2014) with promising results. These authors found that females from a population from Coruripe, Brazil (Brazilian-3 morphotype) were attracted to antennally active pheromone component candidates either used in a synthetic mix or singly. Individual compounds were significantly less attractive in comparison with the blend. In addition, this synthetic blend was equally attractive to females when compared with the volatiles collected from the males, both in laboratory and in field cages. Although the aim of Milet-Pinheiro et al. (2014) research was to find potent female attractants, the same methodology can be applied to evaluate the role of pheromones in mate recognition of closely related species. Similar attractiveness of volatile extracts of calling males and live calling males was reported also by López-Guillén et al. (2011) who found that the number of *Anastrepha obliqua* females captured by traps with volatile extracts of calling males was not significantly different to that caught by live calling males. However, to substitute the natural blend trapped from calling males with synthetics, it is necessary to have access to synthetic compounds (which may not be commercially available and its synthesis may be quite expensive) and to assess technological problems associated with dispenser type(s) that guarantee the pheromone release in comparable concentration and compound ratios like naturally calling males.

## Concluding remarks

Unlike small laboratory cage tests, walk-in field cage tests have shown to be reliable and powerful tools to measure the level of mating compatibility among different species and populations of a putative single species. This experimental arena under semi-natural conditions can also provide good information on the types of pre-zygotic isolation barriers that contribute to reproductive incompatibility. The intermediate scale of field cages, i.e. between small laboratory cages and open field observations, allows this experimental approach to be implemented in several places and without the need of much infrastructure. It also permits the evaluation of the behavior of flies coming from different regions provided adequate bio-security measures are in place (Cayol et al. 2002, Vera et al. 2006, Rull et al. 2012, 2013, Schutze et al. 2013, Devescovi et al. 2014) and, if desired, under different environmental conditions. Minor modifications of the general protocol can contribute to assess the relative importance of different isolation mechanisms, particularly when subtle differences in the time of mating activity are observed. In order to unravel which signals are crucial to recognize mates from their own species and hence avoid cross-mating, other experimental procedures might be necessary.

Walk-in field cages have also shown their usefulness in determining the role of chemical signals in species recognition. The use of calling males ensures the timely release of the compounds at the right proportions while the use of volatile compounds, instead of male flies, allows evaluating populations in which flies are active at different times of the day. Field cage tests can be accompanied by laboratory tests to assess antennal responses in the females to the various compounds present in the male pheromone. In addition, the role of close distance chemical signals such as cuticular hydrocarbons and other non-chemical signals such as acoustic and visual stimuli remains to be further explored.

Within the context of SIT application, the use of walk-in field cages to asses mating compatibility has not been restricted to the Tephritidae. For tsetse flies see Mutika et al. (2001, 2013) and for an evaluation of mating compatibility among codling moth, *Cydia pomonella* (L) populations from different regions of the world see Taret et al. (2010).

Overall, the so far attempted evaluation of different testing conditions has shown to provide a better understanding of the pre-zygotic isolation barriers occurring when mating incompatibility is found. As a general recommendation, the most convenient approach in a novel situation is to start with the traditional protocol and only in those cases in which sexual isolation is associated with temporal isolation, adaptations to the standard protocol should be made to evaluate mating compatibility. The experimental approach reviewed here provides a good source of information to delimit reproductive species boundaries. However, in any case it is advisable to follow this methodology as part of an integrative taxonomy approach, including also molecular, physiological and morphological traits in the assessments in order to achieve robust species delimitation.
